# Prehospital Defibrillation Challenges in Victims Wearing Wetsuits: A Pilot Comparison of AED Pad Placement Strategies

**DOI:** 10.3390/jcm14217536

**Published:** 2025-10-24

**Authors:** Myriam Santos-Folgar, Martín Otero-Agra, David Currás-García, Felipe Fernández-Méndez, Roberto Barcala-Furelos, Antonio Rodríguez-Núñez

**Affiliations:** 1 REMOSS Research Group, Faculty of Education and Sport Sciences, Universidade de Vigo, 36005 Pontevedra, Spain; m.santos.folgar@gmail.com (M.S.-F.); fernandez.mendez.felipe@gmail.com (F.F.-M.); roberto.barcala.furelos@gmail.com (R.B.-F.); 2 School of Nursing, Universidade de Vigo, 36001 Pontevedra, Spain; 3 Department of Obstetrics, Complexo Hospitalario of Pontevedra, Sergas, 36001 Pontevedra, Spain; 4 CLINURSID Research Group, Psychiatry Radiology Public Health Nursing and Medicine Department, Universidade de Santiago de Compostela, 15705 Galicia, Spain; Antonio.Rodriguez.Nunez@sergas.es; 5 Simulation and Intensive Care Unit of Santiago (SICRUS) Research Group, Health Research Institute of Santiago, University Hospital of Santiago de Compostela—CHUS, 15706 Santiago de Compostela, Spain; 6 Collaborative Research Network Orientated to Health Results (RICORS), Primary Care Interventions to Prevent Maternal and Child Chronic Diseases of Perinatal and Developmental Origin, Instituto de Salud Carlos III, 28029 Madrid, Spain; 7 Faculty of Nursing, Universidade de Santiago de Compostela, 15705 Santiago de Compostela, Spain; 8 Paediatric Critical Intermediate and Palliative Care Section, Hospital Clínico Universitario de Santiago de Compostela, Sergas, 15706 Santiago de Compostela, Spain

**Keywords:** defibrillation, rescuer, cardiac arrest, antero-lateral, antero-posterior, wetsuit

## Abstract

**Objective**: This pilot study compared the positions of the antero-lateral (standard) and antero-posterior (alternative) pads in a simulated cardiac arrest scenario in athletes wearing a wetsuit. **Methods**: Seventeen undergraduate physical education students were instructed to attend to a simulated victim, with no signs of life, dressed in a wetsuit. In a randomized fashion, they were instructed to place the defibrillator pads in the standard position (antero-lateral) or in the antero-posterior option. The variables analyzed were the time required to perform the procedure and the difficulty and fatigue perceived by the rescuers. **Results**: Thirty-four interventions were analyzed (17 with each technique), showing that with the antero-posterior option, the time required to expose the area was less (median 6.2 vs. 12.7 s, *p* = 0.001), but more time was required to dry it (median 31.0 vs. 18.4 s, *p* = 0.002). No significant differences were found between the two options in the total time from onset to first flush or in the perception of difficulty and fatigue. **Conclusions**: In the case of caring for a cardiac arrest victim wearing a wetsuit, the alternative of placing the defibrillator pads in the antero-posterior position is not a significant advantage over the standard position. Both configurations may be considered acceptable in prehospital aquatic settings, depending on situational constraints and rescuer preference.

## 1. Introduction

Early defibrillation is essential to increase survival rates in case of cardiac arrest [1,2,3,4], since if performed within the first 3–5 min after collapse, the chances of survival can reach 50–70% [2,3,4]. This time-dependent relationship highlights the need for rapid recognition of cardiac arrest, immediate cardiopulmonary resuscitation (CPR), and prompt defibrillation as part of the chain of survival [1].

Sudden cardiac arrest can occur in any environment and activity [5]. Although most cases occur in non-athletic populations, sports-related sudden cardiac arrest represents a distinct challenge due to the specific environmental conditions, physical demands, and possible delay in emergency response times [6].

Sports activities, amateur or professional, are popular and especially at risk when performed in the aquatic environment [7]. The widespread use of neoprene wetsuits, both in cold and warm water, could hinder some of the cardiopulmonary resuscitation (CPR) maneuvers, such as the rapid and effective placement of semiautomatic defibrillator (AED) electrodes [6].

This becomes particularly relevant in coastal or open-water events such as triathlons, surf competitions, or diving activities, where sudden cardiac arrest may occur in athletes or recreational users wearing wetsuits. In these cases, fast and effective AED pad placement becomes critical to ensure successful defibrillation.

In addition, rescuers may face increased stress in these contexts due to environmental conditions, high turnout of participants, and limited access to the victim, making rapid intervention even more complex.

In this context, given that as an alternative to the antero-lateral position, CPR recommendations include the antero-posterior position, especially in refractory cases and in pediatric patients [8] and that recent studies suggest that the antero-posterior position could have advantages in certain circumstances [9,10] we conducted this study whose objective is to compare both options in a simulated cardiac arrest scenario in a victim dressed in a wetsuit.

However, to date, no clinical guidelines or studies have specifically addressed AED pad placement strategies in cardiac arrest victims wearing wetsuits—an increasingly relevant concern for prehospital and emergency medicine providers.

Therefore, this study aims to compare the efficiency, speed, and ease of AED pad placement techniques in antero-lateral and antero-posterior positions, evaluating them in wetsuit-clad athletes, in order to improve outcomes in cardiac arrest situations.

## 2. Materials and Methods

### 2.1. Sample

Seventeen lifeguards (13 men and 4 women), students of the Degree in Physical Activity and Sport Sciences, participated in the pilot study. All had passed the Lifeguarding and its Didactics course, which includes CPR practice and the use of the AED. Participation was voluntary. The study was approved by the Ethics Committee of the Faculty of Education and Sport Sciences of the University of Vigo (code 12-181223) and complied with the Helsinki ethical principles.

### 2.2. Previous Skills Training

All participants received training in first aid, basic CPR, and AED use, including the two options for electrode placement (antero-lateral and antero-posterior). The training sessions began with a brief theoretical review covering the pathophysiology of sudden cardiac arrest, the chain of survival, and the importance of early defibrillation. This was followed by practical demonstrations where participants practiced pad placement on mannequins, ensuring correct positioning and adhesion according to each configuration. The Laerdal AED trainer (model 197-01050, Laerdal Medical, Stavanger, Norway) was used, and the recommendations of the ERC 2021 were followed [8]. As this is a training device, no real shocks were delivered, and no impedance or electrical variables were measured. During the practical phase, emphasis was placed on minimizing hands-off time, optimizing compression–shock intervals, and adapting pad placement techniques for scenarios involving wetsuit-clad victims. Training included simulated scenarios, but these did not involve neoprene wetsuits. Feedback was provided in real time to correct technique and ensure adherence to ERC guidelines [8].

### 2.3. Study Design

**Figure 1:**
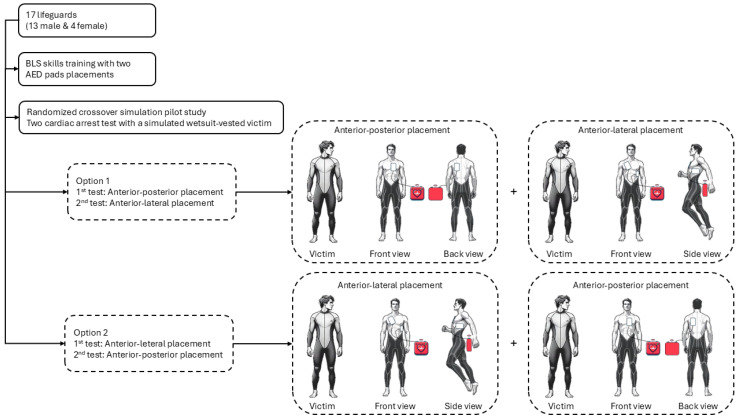
Flow chart.

A pilot, randomized, crossover study was performed (Figure 1) in a simulated cardiac arrest scenario in the aquatic environment. Data collection was conducted at Mogor Beach, Pontevedra, Spain (42°23′08.0″ N 8°43′11.1″ W). The simulated victim, positioned on land after a hypothetical water rescue, wore a full-body neoprene wetsuit with a rear zipper—typical of aquatic sports—which limited anterior chest access and recreated real-life barriers to resuscitation. The scenario included environmental and clothing-related challenges commonly faced during aquatic rescues, such as delayed CPR maneuvers and complications in AED electrode placement. The sequence of scenarios was determined by a computer-generated randomisation list (block size = 2, allocation ratio 1:1) and remained concealed until the time of allocation.

**Figure 2:**
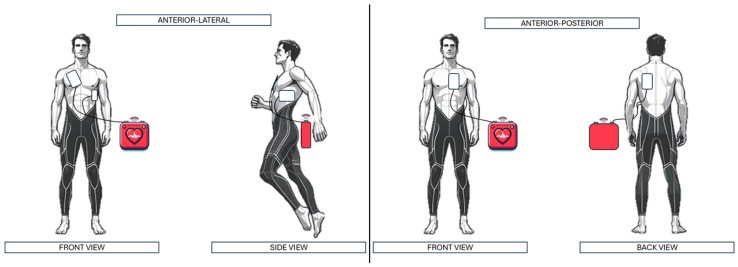
Demonstration of electrode placement in each scenario.

Each rescuer performed two CPR attempts and was instructed to place the AED electrodes in the antero-lateral position (standard position) and antero-posterior position (alternative). In the antero-lateral (AL) position, the right pad was placed in the right infraclavicular region and the left pad on the left lateral chest along the midaxillary line. In the antero-posterior (AP) position, the anterior pad was placed on the sternal/precordial area and the posterior pad on the left interscapular region (Figure 2). The sequence of the tests was randomized. All participants successfully completed both pad placement techniques.

### 2.4. Variables

The following variables were recorded during each simulation:

a. Demographic variables: Baseline demographic characteristics were collected prior to the simulation, including age, sex, height, and weight. Body mass index (BMI) was calculated from these measurements.
b. AED variables: The duration of each step involved in the AED application sequence was recorded in seconds, from the start of the scenario to the delivery of simulated defibrillation. The process was divided into the following segments:
  - T1: From the start of the scenario to the point at which the wetsuit was sufficiently removed to allow drying of the chest and placement of the AED electrodes.
  - T2: From the end of T1 to proper drying of the skin, necessary to ensure effective electrode adhesion and electrical conductivity.
  - T3: From the end of T2 to the placement of the AED electrodes in the assigned position.
  - Total time: From the beginning of the simulation to the delivery of effective defibrillation, representing the cumulative duration of all critical steps.
c. Perceived fatigue and difficulty variables: After each test, participants rated the difficulty of electrode placement on an ordinal scale from 0 (no difficulty) to 10 (extreme difficulty), reflecting their subjective perception of the complexity involved in the assigned position. Additionally, following each attempt, participants reported their level of physical exertion using a modified Borg scale [11], providing a standardized measure of fatigue associated with the procedure.

### 2.5. Statistical Analysis

Different perspectives were taken into account when deciding on the sample size for the study. It was proposed to use a sample size that would allow: (1) evaluation of the feasibility of the protocol; (2) obtaining preliminary estimates of the magnitude and variance of the differences between the two placements; (3) estimating the standard deviation of the differences between the two placements. With all this in mind, it was decided to use n = 17, so that large effects in the differences could be detected. Thus, with an α = 0.05 and a 1 − β = 0.80, an effect size dz = 0.65 was obtained. All sample size calculations were performed using G*Power 3.1.9.2 software (Heinrich-Heine-Universität, Düsseldorf, Germany).

Statistical analysis was performed with IBM SPSS Statistics v.21 for Windows (Armonk, NY, USA). Prior to performing statistical analyses, an analysis was conducted to assess period effects based on the order of the two tests performed, which is shown in Table S1. Following this, the categorical variables were described using absolute and relative frequencies. Continuous variables were described using measures of central tendency (median) and dispersion (IQR: interquartile range). The Wilcoxon test or Student’s *t*-test was used to analyze the variables, depending on whether or not they met the criteria for normality (Shapiro–Wilk test). A significance level of *p* < 0.05 was established. Cohen’s test [12] or Rosenthal’s test [13] was performed in accordance with the normality criteria to calculate the effect size (ES). To describe the effect size, Rosenthal’s r was used for the following classifications: small (0.1–0.3), moderate (0.3–0.5), large (>0.5). For Cohen’s d, the following classifications were used: small (0.2–0.5), moderate (0.5–0.8), large (0.8–1.3), and very large (>1.3).

## 3. Results

### 3.1. Demographic Variables

The results of the demographic variables are shown in Table 1. A total of 13 (76%) of the 17 total participants were male. The sample had a median age of 22 years (IQR: 20–22), a median weight of 75 kg (IQR: 67–80), and a median height of 174 cm (IQR: 170–176).

### 3.2. AED Variables

The results of the AED variables are shown in Table 2. A significantly shorter time from test start to neoprene removal was observed with the antero-posterior placement (6.2 s; IQR: 4.9–7.1) than with the antero-lateral placement (12.7 s; IQR: 10.4–15.0), with a *p*-value = 0.001 and an ES = 0.83. On the other hand, a significantly shorter time from neoprene removal to skin drying was observed with the antero-posterior placement (31.0 s; IQR: 25.1–34.9) than with the antero-lateral placement (18.4 s; IQR: 15.4–23.8) with a *p*-value = 0.002 and an ES = 1.20. No differences were observed in the time from skin drying to electrode placement (antero-posterior: 36.3 s; IQR: 31.3–41.4/antero-lateral: 32.8 s; IQR: 27.9–38.7/*p* = 0.10). There were also no differences in the total time of the whole test (antero-posterior 74.0 s; IQR: 40.4–81.8/antero-lateral: 63.5 s; IQR: 55.2–69.6/*p* = 0.52).

**Figure 3:**
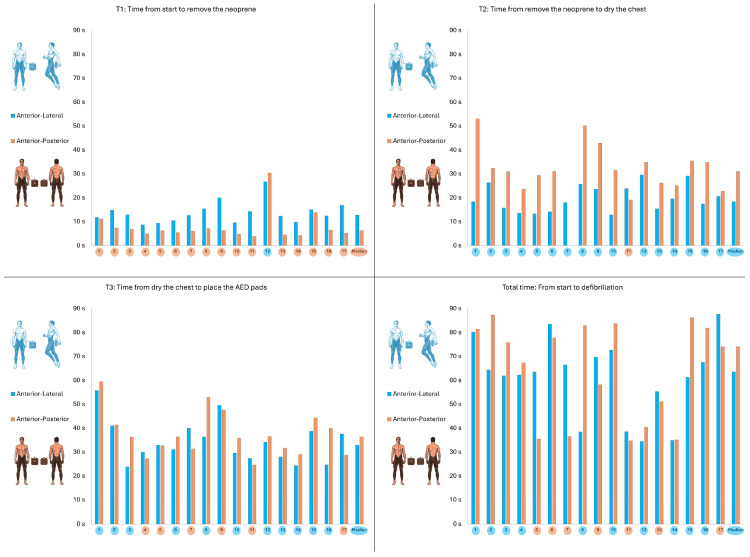
Individual times with antero-lateral placement (blue) and antero-posterior placement (orange).

Figure 3 shows the placement times at each time point and in the total test for each participant.

### 3.3. Perceived Fatigue and Difficulty Variables

The results of the perceived fatigue and difficulty variables are shown in Table 3. The antero-posterior placement reflected a higher perceived fatigue than the antero-lateral placement, reflecting in both cases a slight level of fatigue (antero-posterior: 1; IQR: 0–2/antero-lateral: 1; IQR: 0–1/*p* = 0.046; ES = 0.49). No differences were observed in the perceived difficulty between antero-posterior and antero-lateral placements.

## 4. Discussion

The primary objective of this pilot study was to compare the efficiency, speed, and ease of antero-lateral versus antero-posterior AED pad placement in a simulated aquatic rescue scenario involving a victim wearing a wetsuit. The main finding was that both configurations were feasible and did not significantly differ in total time to shock delivery, perceived difficulty, or fatigue.

In the event of having to resuscitate a victim dressed in a wetsuit, the placement of the AED patches may delay and/or hinder the procedure, and no specific guidelines are available for their placement in such a situation. If we extrapolate the current recommendations to that scenario, both the classic option (antero-lateral position) and the alternative (antero-posterior position) could be indicated [8]. Our results support the feasibility of the antero-posterior configuration in aquatic rescue contexts, showing that it can be applied without a significant impact on total time to shock delivery. These findings are consistent with previous studies [14,15,16] that have also supported the use of the antero-posterior position, particularly in improving shock efficacy and reducing transthoracic impedance. Although those studies focused on physiological parameters rather than procedural timing, the concordance reinforces the clinical value of this alternative configuration, especially when anterior chest access is delayed or limited by wetsuit design.

On the other hand, our results showed a significantly longer drying time (T2) for the antero-posterior placement compared to the antero-lateral position. Drying the chest prior to AED pad placement is a critical step in aquatic cardiac arrest scenarios to ensure optimal pad adhesion and minimize transthoracic impedance, thereby improving shock efficacy [17]. Moisture on the skin can hinder electrode adherence, increase electrical resistance, and potentially reduce the likelihood of successful defibrillation. In addition, although studies suggest that AED use in wet environments does not increase the risk to the rescuer or patient in the absence of direct contact [18], clinical guidelines still advise ensuring that pads are applied to dry skin [19]. In our study, the antero-posterior configuration required a longer chest-drying time—likely due to the need to dry two distinct skin areas for pad placement. However, this expected difference did not translate into a longer total time to shock delivery, indicating that the additional drying time did not negatively affect overall procedural efficiency. This finding reinforces the feasibility of the antero-posterior position in wetsuit-clad victims, even when accounting for the extra step of drying multiple contact sites.

Although the additional drying time did not affect the total time to shock delivery, the success of defibrillation depends on more than speed alone. Factors such as pad adhesion [19], patient positioning, anatomical variations, and chest access [10] may influence shock efficacy. For this reason, training programs for lifeguards and Emergency Medical Services providers should emphasize proficiency in both configurations, enabling adaptation to different wetsuit types (full-body, varying thicknesses, rear or front zippers, or no zipper) and operational contexts. This adaptability could help reduce delays without compromising effectiveness.

From a prehospital care perspective, these findings are relevant for emergency medical personnel, lifeguards, and first responders who may face cold temperatures, moisture, unstable surfaces, and limited chest access in aquatic rescues [18]. The antero-posterior configuration might be preferred when removing the wetsuit anteriorly is not feasible or is excessively time-consuming, as in full-body suits with rear zippers or integrated buoyancy systems. Moreover, considering the scarcity of controlled evidence to guide resuscitation protocols in drowning-related cardiac arrest scenarios, as highlighted by Bierens et al. [20], incorporating both techniques into standard aquatic rescue training could strengthen preparedness and decision-making in the field.

Furthermore, in real-life cardiac arrest cases, where the chances of survival decrease rapidly with every minute of delay in defibrillation [21], any enhancement in the speed or fluidity of the procedure could translate into improved survival outcomes. While our results showed no statistically significant difference in total time to shock, understanding the variability introduced by suit characteristics and rescuer actions could help optimize protocols and training.

This pilot study also raises several questions that remain unanswered, such as how results might differ with more experienced rescuers or actual cardiac arrest patients, whether wetsuit characteristics (thickness, zipper placement) influence performance, and how environmental stressors like cold water, unstable surfaces, or physical exhaustion may impact efficiency. These aspects warrant further research to better understand the clinical implications of AED pad placement in aquatic emergencies.

Finally, this study highlights the need for formal clinical guidelines or operational recommendations addressing AED use in special conditions, such as wetsuit-wearing athletes, to bridge the gap between evidence and field practice.

Future studies should further investigate how wetsuit design—such as neoprene thickness, zipper placement, or chest coverage—affects pad placement and shock delivery, and whether findings extend to dry suit scenarios in cold-water diving. It would also be useful to compare different AED pad models and explore potential device modifications (e.g., pads with enhanced adhesion, rapid-dry surfaces, or alternative geometries) to optimize resuscitation in aquatic environments.

Limitations of this pilot study include the small sample size and the fact that the participants were university students with previous lifeguarding experience, so it is possible that the lay subjects had more difficulty or required more time to perform the procedures investigated. Although the training phase included practice, these scenarios did not involve actual neoprene wetsuits; therefore, participants applied AED pads on wetsuit-clad victims for the first time during the study trials. On the other hand, the design with simulation in a controlled environment may not accurately reflect the challenges posed by real situations. In real out-of-hospital cardiac arrest scenarios, especially in aquatic or outdoor environments, additional clinical variables may interfere with the procedure, such as water temperature affecting the rescuer’s motor skills, wet or unstable surfaces, physical exhaustion, the presence of bystanders or distractions [17,22]. Moreover, the study did not consider the psychological and physiological stressors inherent in real emergencies, such as time pressure, emotional stress, or multitasking demands, which could significantly influence performance. Finally, the study did not evaluate the electrical efficacy or impedance of shock delivery, nor did it assess electrode adhesion or survival-related outcomes, all of which are critical to the clinical applicability of AED pad placement strategies. Additionally, only one training AED model was used, which may limit the generalizability of our findings to other devices.

## 5. Conclusions

In the case of caring for a cardiac arrest victim wearing a wetsuit, no statistically significant differences were observed between antero-posterior and antero-lateral pad placement in terms of time to shock delivery or perceived difficulty. Preliminary data suggest that both configurations appear feasible and may be used depending on the context, rescuer experience, and the characteristics of the wetsuit.

Our results indicate potential flexibility of using either pad placement technique depending on the situation, the characteristics of the wetsuit, and the rescuer’s experience. The anterior–posterior position could be particularly useful when anterior chest access is limited or delayed.

Although no significant time differences were found, incorporating both techniques into training programs may help prepare rescuers for real-life scenarios. However, these findings are based on time and process-related outcomes under simulated, land-based conditions using a training AED. Further research is needed to explore the electrical effectiveness of each configuration and to establish evidence-based recommendations for AED use in aquatic environments.

## Figures and Tables

**Table 1:** Demographic variables (n = 17).

Continuous Variables	Median	IQR
Age (years)	22	(20–22)
Weight (kg)	75	(67–80)
Height (cm)	174	(170–176)
Body mass index (kg/m^2^)	24.4	(22.5–26.2)
Categorical Variables	n	(%)
Sex		
Male	13	(76%)
Female	4	(24%)

IQR: Interquartile range; n: absolute frequency; (%): relative frequency.

**Table 2:** AED variables (n = 17).

AED Time Variables	Antero-Lateral Placement	Antero-Posterior Placement	Paired Differences and Their 95%CI	*p*-Value	Effect Size and Its 95%CI
T1: Time from start to remove the neoprene	12.7 (10.4–15.0) 13.7 ± 4.5 (11.4–16.0)	6.2 (4.9–7.1) 7.9 ± 6.3 (4.7–11.2)	Md: 5.9 (3.7–7.9) md: 5.8 (3.6–8.0)	*p* = 0.001 * -	r = 0.83 (0.59–0.94) - -
T2: Time from T1 to dry the chest	18.4 (15.4–23.8) 19.8 ± 5.6 (17.0–22.7)	31.0 (25.1–34.9) 30.8 ± 12.1 (24.6–37.0)	Md: −10.8 (−17.4–−5.5) md: −10.9 (−17.0–−4.8)	*p* = 0.004 * p = 0.002 †	r = 0.71 (0.34–0.89) d = 0.92 (0.26–1.54)
T3: Time from T2 to place the AED pads	32.8 (27.9–38.7) 34.4 ± 8.8 (29.8–38.9)	36.3 (31.3–41.4) 37.4 ± 9.37 (32.6–42.2)	Md: −3.7 (−5.6–1.9) md: −3.0 (−6.7–0.7)	*p* = 0.102 * p = 0.103 †	r = 0.40 (−0.10–0.74) d = 0.42 (−0.96–0.12)
Total time: from start to shock	63.5 (55.2–69.6) 61.3 ± 16.4 (52.8–69.7)	74.0 (40.4–81.8) 64.1 ± 20.7 (53.4–74.7)	Md: −1.1 (−13.9–5.7) md: −2.8 (−12.4–6.9)	*p* = 0.523 * -	r = 0.15 (−0.35–0.59) - -

Results described using median and interquartile range (top) and using mean ± standard deviation and 95% confidence interval (bottom); AED: Automated External Defibrillator. Md: Median differences; md: Mean differences. Times have been described in seconds. * Signed-rank Wilcoxon test for related samples was performed for comparisons, and Rosenthal’s r was performed for Effect Size. † Student *t* test for related samples was performed for comparisons, and Cohen’s d was performed for Effect Size.

**Table 3:** Perceived fatigue and difficulty variables (n = 17).

Perceived Variables	Antero-Lateral Placement	Antero-Posterior Placement	Paired Differences	*p*-Value	Effect Size and Its 95%CI
Perceived fatigue	1 (0–1) 0.6 ± 0.6 (0.3–0.9)	1 (0–2) 0.8 ± 0.8 (0.4–1.3)	Md: 0.0 (0.0–0.0) md: −0.2 (−0.5–−0.1)	*p* = 0.046 * -	r = 0.49 (0.00–0.78) - -
Perceived difficulty	1 (1–3) 2.1 ± 1.7 (1.2–2.9)	2 (1–4) 2.8 ± 1.9 (1.8–3.8)	Md: −1.0 (−2.0–1.0) md: −0.8 (−1.7–0.1)	*p* = 0.096 * -	r = 0.40 (−0.10–0.74) - -

Results described using median and interquartile range (top) and using mean ± standard deviation and 95% confidence interval (bottom); AED: Automated External Defibrillator. Md: Median differences; md: Mean differences. Times have been described in seconds. * Signed-rank Wilcoxon test for related samples was performed for comparisons, and Rosenthal’s r was performed for Effect Size.

## Data Availability

The original contributions presented in the study are included in the article further inquiries can be directed to the corresponding author.

## Supplementary Materials

The following supporting information can be downloaded at: https://www.mdpi.com/article/10.3390/jcm14217536/s1, Table S1: Testing for period effects (sequence x placement interaction).
