# Supplementary material for: Prehospital Defibrillation Challenges in Victims Wearing Wetsuits: A Pilot Comparison of AED Pad Placement Strategies
Source: J Clin Med. 2025 Oct 24;14(21):7536. doi: 10.3390/jcm14217536 (PMC12607994; doi:10.3390/jcm14217536)
Supplement: Supplementary file 1 [file jcm-14-07536-s001.zip › jcm-3819396-supplementary.pdf]

Supplementary table: Testing for period effects (sequence x placement interaction)

Variables	n = 9 First placement: Anterior-lateral (AL)	n = 8 First placement: Anterior-posterior (AP)	p-value
Sex			
Male	7 (78 %)	6 (75 %)	p = 0.89 ǂ
Female	2 (22 %)	2 (25 %)	
Age	22 (20 – 22)	22 (21 – 22)	p = 0.88 *
Weight	75 (68 – 80)	75 (63 – 85)	p = 0.96 * / p = 0.62 †
Height	173 (170 – 179)	175 (168 – 176)	p = 0.96 * / p = 0.84 †
Body mass index	24.4 (23.2 – 26.2)	24.4 (22.3 – 26.9)	p = 0.85 * / p = 0.48 †
T1: Time from start to remove the neoprene in AL placement	12.8 (12.4 – 15.0)	11.4 (9.7 – 15.1)	p = 0.44 *
T2: Time from T1 to dry the chest in AL placement	18.4 (15.7 – 23.6)	18.5 (13.9 – 26.0)	p = 0.92 * / p = 0.97 †
T3: Time from T2 to place the AED pads in AL placement	37.5 (27.9 – 39.9)	30.5 (27.1 – 35.2)	p = 0.34 * / p = 0.20 †
Total time: from start to shock in AL placement	63.5 (61.2 – 69.6)	63.2 (36.7 – 70.1)	p = 0.56 * / p = 0.35 †
Perceived fatigue in AL placement	0 (0 – 1)	1 (0 – 1)	p = 0.36 *
Perceived difficulty in AL placement	1 (1 – 3)	2 (1 – 3)	p = 1.00 *
T1: Time from start to remove the neoprene in AP placement	6.2 (5.3 – 6.9)	5.9 (4.8 – 7.2)	p = 0.92 *
T2: Time from T1 to dry the chest in AP placement	29.3 (22.8 – 35.5)	31.9 (28.1 – 34.9)	p = 0.44 * / p = 0.51 †
T3: Time from T2 to place the AED pads in AP placement	32.7 (31.3 – 44.3)	36.4 (32.4 – 40.7)	p = 0.70 * / p = 0.99 †
Total time: from start to shock in AP placement	58.1 (36.5 – 75.7)	79.8 (53.8 – 83.3)	p = 0.21 *
Perceived fatigue in AP placement	0 (0 – 2)	1 (0 – 2)	p = 0.76 *
Perceived difficulty in AP placement	3 (2 – 4)	2 (1 – 4)	p =0.43 * / p = 0.38 †

Results described by absolute and relative frequencies (sex) and median and interquartile range.
ǂ Chi Square test
* Mann-Whitney U test for independent samples
† t-test for independent samples
